# Resolving complex structural variants via nanopore sequencing

**DOI:** 10.3389/fgene.2023.1213917

**Published:** 2023-08-16

**Authors:** Simone Romagnoli, Niccolò Bartalucci, Alessandro Maria Vannucchi

**Affiliations:** CRIMM, Center of Research and Innovation of Myeloproliferative Neoplasms, DENOTHE Excellence Center, Careggi University Hospital and Department of Experimental and Clinical Medicine, University of Florence, Florence, Italy

**Keywords:** long read, structural variant, nanopore sequencing, medical genetics, bioinformatics, pipeline

## Abstract

The recent development of high-throughput sequencing platforms provided impressive insights into the field of human genetics and contributed to considering structural variants (SVs) as the hallmark of genome instability, leading to the establishment of several pathologic conditions, including neoplasia and neurodegenerative and cognitive disorders. While SV detection is addressed by next-generation sequencing (NGS) technologies, the introduction of more recent long-read sequencing technologies have already been proven to be invaluable in overcoming the inaccuracy and limitations of NGS technologies when applied to resolve wide and structurally complex SVs due to the short length (100–500 bp) of the sequencing read utilized. Among the long-read sequencing technologies, Oxford Nanopore Technologies developed a sequencing platform based on a protein nanopore that allows the sequencing of “native” long DNA molecules of virtually unlimited length (typical range 1–100 Kb). In this review, we focus on the bioinformatics methods that improve the identification and genotyping of known and novel SVs to investigate human pathological conditions, discussing the possibility of introducing nanopore sequencing technology into routine diagnostics.

## Introduction

The technical advantages provided by massive parallel sequencing made such technology available to worldwide laboratories; hence, next-generation sequencing (NGS) is now a standard for many applications in basic and clinical biology, speeding up the identification of disease-causing genes (Gilissen et al., 2014). NGS technologies contributed to breakthroughs in scientific discoveries, shedding light on the biological context of disease mechanisms. Resequencing of candidate genes or genomic regions of interest in paired samples of affected and/or germline cellular sources, patients, and healthy controls is of key importance to identify pathologic mutations and inherited variants. Resequencing techniques aim at testing known mutations (genotyping) or scanning new mutations in a specific target region (variation analysis). Sequencing data that pass the quality filters are further used as inputs for aligning reads to the reference genome that is crucial for sample genotyping. To this purpose, several read alignment algorithms were developed to map sequencing reads to an existing genome reference. The aligned data are then inspected by variant-callers to detect single-nucleotide variants (SNVs), known as substitutions or point mutations, and other frameshift mutations, in which one or more nucleotides are either added or missing. The typical structure of insertion or deletion (indel) could make their alignment to the reference genome challenging, often resulting in under-detection; to overcome these challenges, paired-end reads were implemented in the NGS workflow and constitute one of the most important technical improvements that facilitates the detection of such abnormalities. Paired-end read sequencing was adopted by NGS platforms (e.g., Illumina) in order to boost the quality of data by analyzing both ends of the same fragments using a second set of reads with opposite orientation with respect to the first set generated. The paired-end approach also facilitates the identification of other genomic rearrangements, such as duplications or amplifications, large deletions, or more complex rearrangements, such as translocations and inversions.

**FIGURE 1:**
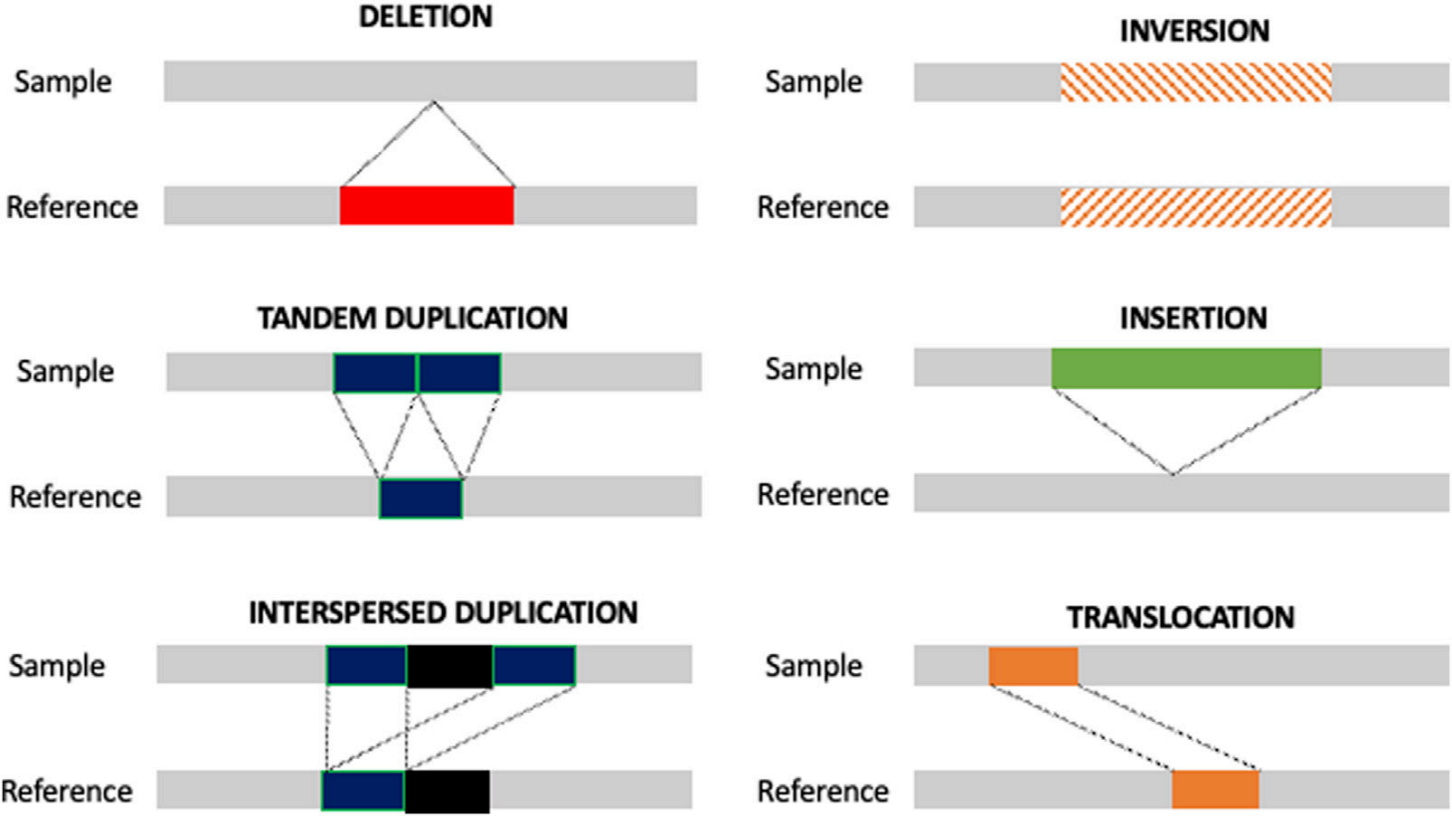
Type of SVs. Schematic representation of the different types of SVs. Unbalanced SVs are represented by deletion, insertion, tandem duplication, and interspersed duplication. Balanced SVs are represented by inversion and translocation.

Although SNVs and small indel were initially believed to contribute to the majority of genomic variations in humans, recent improvements in identifying previously intractable DNA sequences, as well as progress in the human genome assembly, led to the increased importance of structural variants (SVs) in human genetics (Chaisson et al., 2015a; Chaisson et al., 2015b; Seo et al., 2016). SVs are genomic rearrangements longer than 50 bp, including insertions, tandem duplications, interspersed duplications, inversions, and translocations, as well as copy number variants (CNVs) (Figure 1). Unlike SNVs and small indels, SVs can extend to well over megabases of sequences, accounting for more varying base pairs than any other class of sequence variants (Ho et al., 2019). SVs are involved in and, eventually, the driver of several pathologic conditions and hereditary disorders, such as cognitive (Rovelet-Lecrux et al., 2005) and prenatal disorders (Allyse et al., 2015), obesity (Walters et al., 2013), and cancer (Li et al., 2020).

Although SVs are believed to play a major role in the pathobiology and phenotype of different disorders, they have been largely understudied, mostly because their identification is hindered by technical challenges intrinsic to short-read-based technologies (Norris et al., 2016); this holds true, especially for repeated DNA elements, in particular, in low-complexity regions, which are known to be SV hotspots (Mills et al., 2011). In this scenario, third-generation sequencing (TGS) technologies emerged rapidly as powerful tools capable of providing the read length that exceeds several kilobases, thereby overcoming the limitations of the NGS approach (van Dijk et al., 2018). The first commercially available TGS platforms were produced in chronological order by Pacific Biosciences (PacBio) and Oxford Nanopore Technologies (ONT). This review focuses on the detection of complex SVs in the human genome by long-read sequencing technology (ONT) and their contribution to decipher complex human diseases.

### Patterns of SVs

The typical genetic variants studied by high-throughput sequencing technology are represented by SNVs, small insertion/deletions (<50 bp), and SVs. SVs significantly differ from others in type and size and can be broadly categorized into deletions, duplications, inversions, insertions, and translocations. Deletions and duplications are also referred to as CNVs and always characterized by the loss or gain of genomic material; inversions and translocations could be neutral and are referred to as balanced. Several whole-genome analyses revealed that SVs were usually not acquired as one independent event but rather acquired together in patterns (Yi and Ju, 2018). There are several potential mechanisms leading to complex phenomena.

**FIGURE 2:**
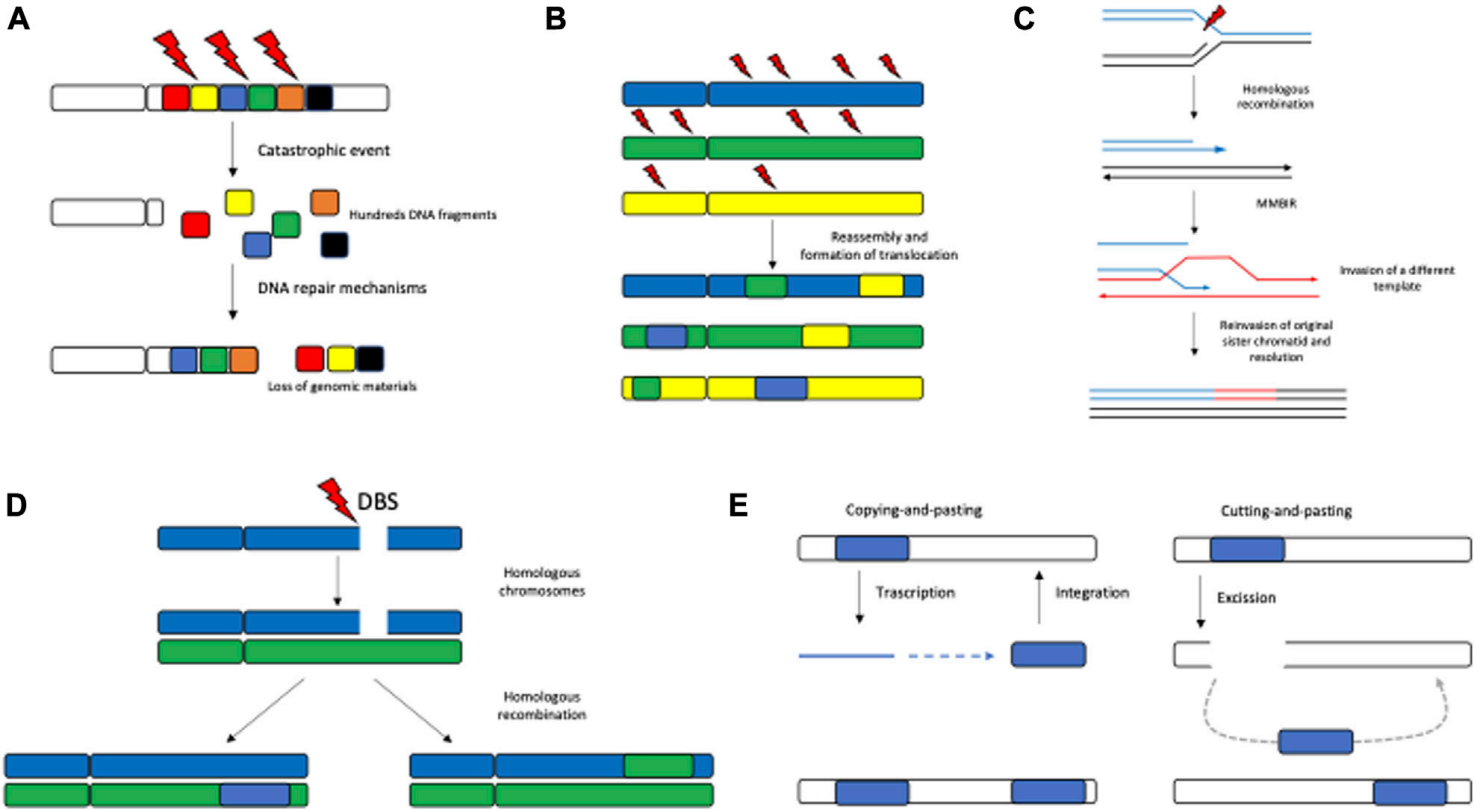
Pattern of SVs. The most recurrent SV patterns are represented by chromothripsis **(A)**, chromoplexy **(B)**, microhomology-mediated break-induced replication **(C)**, homologous recombination **(D)**, and retrotransposable elements **(E)**.

Chromothripsis is a phenomenon in which one or a few chromosomal arms are affected by multiple chromosomal rearrangements. This particularly occurs in osteosarcoma, chordoma (∼25%), and brain tumors (∼10%) and is overall found in 3% of tumors. The term chromothripsis originates from the Greek words *chromo* for “chromosome” and *thripsis* meaning “shattering into pieces.” The Greek term perfectly illustrates the events occurring when one or few chromosome arms are shattered into hundreds of DNA segments simultaneously and the DNA repair machinery reassembles the fragments in an incorrect order and orientation. Cancer cells are characterized by increased chromosome instability that also manifests with chromothripsis. The chromosomal regions involved in chromothripsis show a massive number of balanced chromosomal rearrangement types such as deletion, tandem duplication, and head-to-head and tail-to-tail inversions (Forment et al., 2012) (Figure 2A).

Chromoplexy differs from chromothripsis in which multiple SVs are localized in one or few chromosome arms; however, such SVs mainly constitute interchromosomal translocations. This mechanism, which might result in the disruption of tumor suppressor genes and activation of oncogenes, is mostly common in prostate cancer (∼90%) (Baca et al., 2013). A chromoplexy event usually involves more than three chromosomes, and, even if small deletions may be present around the breakpoints, it is generally considered a copy number neutral alteration (Figure 2B).

Gains of genomic materials are relatively infrequent in both chromothripsis and chromoplexy; however, in cancer and pathologic non-neoplastic genomes, interspersed copy number gains of one parental allele are frequently observed, without evidence of loss of heterozygosis (LOH) (Rovelet-Lecrux et al., 2005).

Microhomology-mediated break-induced replication (MMBIR) is a feature by which a cell gains genomic materials, switching on the replication machinery. The molecular basis of MMBIR is unclear; nevertheless, persistent replication stress involving *Rec*/*RAD* is reported to trigger MMBIR through collapsing the replication fork due to a single-strand DNA break interfering with normal DNA replication, stimulating template switching (Hastings et al., 2009) (Figure 2C).

Another recurrent pattern of SVs is the homologous recombination (HR) repair defect, leading to increased genome instability, as it involves the machinery of double-strand breaks. HR was described in breast and ovarian cancer and leads to the complete inactivation of *BRCA1* and *BRCA2*. Specific patterns of SVs, in particular, short tandem duplications and deletions, were found in *BRCA1* and *BRCA2* cancer genomes, respectively (Nik-Zainal et al., 2016) (Figure 2D).

SVs are also found to be localized in the region rich in repetitive elements, which represents as much as 45% of the human genome (Craig Venter et al., 2001). In these regions, SVs were generated by transposable elements via “cutting-and-pasting” (DNA transposons) or “copying-and-pasting” themselves (retrotransposons) (Figure 2E). Retrotransposition elements were found in the heterochromatin and hypomethylated regions, leading to aberrant gene expression (Yi and Ju, 2018).

### Long-read vs. short-read sequencing technology: risks and advantages

**FIGURE 3:**
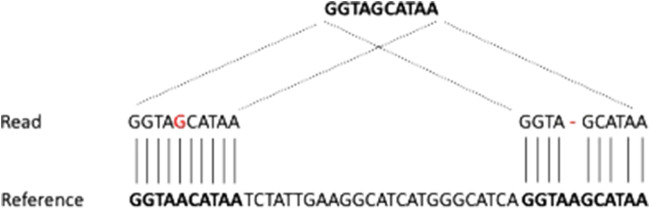
Ambiguities in read mapping. A read mapping equally well to two different locations is assigned to either the first or the second location depending on the score given by the aligner to mismatches and gaps.

NGS approaches enabled the investigation of genomic regions at a base resolution level and allowed the discovery of novel molecular abnormalities, fostering considerable progress, not only in the understanding of disease pathogenesis but particularly in the development of translational diagnostic assays, and making the rationale for the development of novel therapies. WES and targeted sequencing provided by NGS are powerful and cost-effective tools to investigate candidate variants occurring in coding regions through the employment of a sequencing panel that targets all the coding sequences of genes or specific regions of interest. On the other hand, it is extremely difficult to attribute pathogenicity to a variant occurring outside the coding regions. NGS approaches allowed the evaluation of the mutational status of the specific gene of interest related to the patients’ phenotype in approximately one-third of rare genetic diseases (Clark et al., 2018). In the remaining two-thirds of the cases, the apparent lack of characterization might be due to the localization of the pathogenetic mutations in an inaccessible region by conventional NGS approaches, e.g., in repetitive regions of GC-rich regions where SVs are known to be mostly located. In many instances, the molecular landscape of the disease remains poorly understood since an informative view of genomic variants was not fully provided by conventional approaches and no candidate variants in coding regions could be identified (Gilissen et al., 2014; Miller et al., 2021a). The introduction of the paired-end strategy boosted the ability to detect complex rearrangements by exploiting the localization of the pairs, as well as the coverage at the SV breakpoint. In the case of duplications and deletions, the number of reads around the resulted breakpoints increased and decreased, respectively. On the other hand, in complex rearrangements, the pairs are abnormally oriented and do not affect the coverage. Overall, the detection of SVs by short-read sequencing is an efficient method to search for most known SVs; conversely, classical NGS approaches often fail to detect novel SVs (Goodwin et al., 2016), especially for insertions (Chaisson et al., 2015a; Sedlazeck et al., 2018; Audano et al., 2019), mainly because they are limited by a low resolution of repeated sequences (Delaneau et al., 2013). When aligned to the reference genome, a repeated region could be erroneously collapsed on top of one another, causing complex, misassembled rearrangements (Treangen and Salzberg, 2012; Mantere et al., 2019). Overall, the repeat content in the human genome is estimated at ∼50%, contrasting with the percentage of short-read mapping to a unique region in the human genome that is typically reported to be ∼80%, although such an estimate varies depending on the read length, the approach used (e.g., single-end reads or paired-end reads), and the performance of the aligners (Treangen and Salzberg, 2012). This discrepancy mainly depends on the non-exact nature of most repeats, which implies that they will have a unique best match even if the same sequence occurs with slight variations in another location. The simplest way to resolve repeats may be the alignment of the reads to the best position; however, this may not always be appropriate due to the presence of different targets where the reads are supposed to map to. In all instances, in those cases where the read may map to region A with one mismatch and to region B with one deletion, the performance of the aligner is crucial, e.g., if the aligner considers mismatches more likely to occur than deletions, the read would be mapped to location A. However, if the source DNA has a true deletion, the read would perfectly match position B. This issue, which is inherent in the process of aligning reads to a reference genome, is shown in Figure 3. This circumstance happens not only in the context of repeated elements but also whenever a given read maps to multiple locations. This phenomenon is highly relevant when attempting to detect translocations. From a computational point of view, a translocation is characterized by reads that map in two different portions of the genome (chromosome) and are also referred to as chimeric reads or split reads. TGS is expected to facilitate the resolution of the translocation by improving the alignment of chimeric reads that, given the length of typical reads, usually span the SV breakpoints.

In line with the aforementioned overview, long-read sequencing approaches also demonstrated the key relevance of the phasing of haplotypes (the process of the estimation of haplotypes, maternal or paternal, from genotype data). The availability of long reads encompassing multiple variant breakpoints, including SNVs and SVs, facilitates the phasing of multiploidy genomes, as well as other haplotype-resolved analysis (Wang et al., 2021; Tewhey et al., 2011).

Read mapping is a crucial step in characterizing SVs because, unlike SNPs and small indels, structural alterations may result in larger reads than the short read generated by conventional NGS with several orders of magnitude; for this reason, long reads that cover a huge portion of a complex sequence facilitates the reconstruction of the SV. For example, a large insertion constitutes an increment in the base content of a given sequence and a corresponding lack in the reference genome; consequently, if a given read fails to align before and after the insertion, it is discarded, making the identification unreliable. In the case of a deletion, it leads to a larger insert size (the distance of the pairs) (Mahmoud et al., 2019).

Moreover, SVs often induce similar or complex mapping patterns, making it more difficult to distinguish tandem duplications from novel insertions for genomic alignments or multiple SVs nested together. TGS technologies have the potential to characterize those genomic elements which are problematic for conventional NGS-based approaches, e.g., by identifying SVs and repeat expansion in regions extremely rich in GC and by revealing chromosomal contexts in which disease-causing mutations are harbored (Miller et al., 2021; Sakamoto et al., 2021). TGS is mostly applied to study complex alterations affecting wide genomic regions (Jain et al., 2018) and *de novo* assembly, where the long nature of typical TGS reads increases the potentiality to identify SVs with higher precision, especially for genomic SVs spanning several kilobases or enriched in repetitive elements, and allows the assembly of contigs spanning several nucleotides, also exploited as a scaffold for the assembly of shorter contigs.

In addition to NGS and TGS, conventional cytogenetics and array-based technology, such as microarray comparative genomic hybridization, are traditionally exploited for the identification and characterization of SVs.

Cytogenetics, which includes karyotyping and fluorescence *in situ* hybridization (FISH), allows the detection of genetic biomarkers of diseases. Although cytogenetics is a standard assay, this method faces limitations due to the resolution of ∼5 Mb, and as for FISH, the *a priori* knowledge of which loci to test is limited in throughput (Neveling et al., 2021).

Array-based technologies, such as array comparative genomic hybridization (aCGH), can detect only a certain type of SVs and are not suitable for mapping small or copy-balanced SVs as well as for resolving tandem duplication from insertion *in trans* (Alkan et al., 2011; Kosugi et al., 2019). Conversely, the long read provided by ONT has the potential to fully cover the SV breakpoints, providing reads with no theoretical limit in terms of read length (Huddleston et al., 2017).

A considerable effort in the identification and characterization of SVs could be provided by the so-called “optical genome mapping” (OGM). OGM provides a single, cost-effective method with a significantly high resolution compared to conventional karyotyping, FISH, and array-based technologies. OGM is used to reconstruct the genome with a highly accurate structure and contiguity in consensus maps up to chromosome arm length. Label pattern differences relative to a reference are detected, and these differences are used to call SVs (Neveling et al., 2021).

Genome imaging of an extremely long linear molecule offered by OGM has the potential to replace all three aforementioned assays in diagnostic procedures. Moreover, taking into the account the higher accuracy of OGM in the identification and characterization of SVs and CNVs, this approach has the potential to be used as an orthogonal validation of SVs identified by long-read sequencing technologies.

As demonstrated, SVs play a prominent role in the development of several human neoplasms, although such types of alterations are largely understudied and represent a technical challenge for conventional sequencing technologies (Huddleston et al., 2017; de Coster et al., 2019). Furthermore, Huddleston et al. (2017) showed that approximately 89% of human variations consist of SVs, and those were missed as part of the analysis of the 1000 Genomes Project. In this study, authors identified signatures of putative SVs from the alignments on CHM1 and CHM13 via single-molecule real-time (SMRT) long-read sequencing. They found 20,602 SVs in CHM1 (12,998 insertions, 7,557 deletions, and 47 inversions) and 20,470 SVs in CHM13 (13,118 insertions, 7,306 deletions, and 46 inversions), of which 83% (17,019/20,602) SVs in CHM1 and 83% (16,939/20,470) SVs in CHM13 were previously unreported. They also merged CHM1 and CHM13 datasets into a theoretical diploid genome, identifying 30,062 SVs corresponding to 13.4 Mbp of sequence difference between the two genotypes. It is interesting to note that this single synthetic diploid recapitulates more than 40% of the SVs that were previously reported in Phase 3 of the 1000 Genomes Project, and that 89% of them were previously missed in the short-read genomes (Sudmant et al., 2015).

## Oxford Nanopore Technology

ONT developed the first sequencing technology that uses a nanopore as a biosensor to sequence long DNA molecules. The first commercially released ONT sequencing device was the MinION, a pocket-sized cost-affordable instrument producing high-throughput sequencing data in real time (Ip et al., 2015) (https://nanoporetech.com/products/minion).

Each nanopore hosted in the flow cell is connected to an electrode and a sensor chip (called application-specific integrated circuit (ASIC)) that measures the electric current flowing through the nanopore channel. As the DNA molecules translocate through the pore, different nucleotide combinations create a characteristic disruption in the ionic current, also referred to as “squiggle.” The observed shift in the ionic current is not influenced by a single nucleotide but rather by k-mers. A k-mer is a subsequence of length k part of a nucleic acid strand; ONT exploits the signal derived from 5-mers (Lu et al., 2016). When a DNA molecule comes in close proximity to the pore, a helicase enzyme unwinds the paired double strands and fosters the translocation of a single strand through the pore. The “squiggle” resulting from the passage of the 5-mers composing the strand is then decoded in real time by the basecalling algorithms to output the DNA (RNA or cDNA) sequence. The changes in the ionic currents are also influenced by epigenetically modified bases.

Currently, ONT has released more than eight versions of chemistry contained in the flow cells since 2014. The first version R6 was released in June 2014, while versions R7, R7.3, R9, R9.4, R.5, R10, R10.3, and R10.4 were released in July 2014, October 2014, May 2016, October 2016, May 2017, March 2019, January 2020, and January 2022, respectively.

The read length of nanopore sequencing has no apparent technical limit, but it is highly affected by the quality and fragmentation of the input sample; therefore, nucleic acid extraction is a key step in maximizing the throughput of sequencing. The main drawback of nanopore sequencing is the relatively high error rate (ranging from 5% to 20%) compared to other sequencing technologies. To increase the accuracy, ONT developed a method to sequence both strands of a double-stranded DNA molecule. In this method, called 1D^2^, an adaptor with a specialized sequence promotes the entry of the second strand into the pore after the first strand. The 1D^2^ protocol could increase the accuracy by up to 97% and lower the error rate down to 6.7%. Given that both strands of each molecule are sequenced, the consumption of pores is doubled, and the boost, in terms of accuracy, comes at the cost of a lower sequencing throughput (Silvestre-Ryan and Holmes, 2021).

The accuracy of the raw reads, as well as the sequencing yield per unit per time, has increased with the release of these new systems. With the latest update of nanopore sequencing, the flow cells with chemistry R10.4.1 can achieve an accuracy of ∼99% using the “Super accuracy” basecaller in MinKNOW with a processing speed of ∼400–450 bases per second, although an independent study is needed to assess this claim.

All ONT devices rely on the flow cells, and in addition to the aforementioned chemistry, there are three types of ONT flow cells: the Flongle, the MinION/GridION, and the PromethION flow cells. First, the Flongle, which is compatible with both MinION and GridION, can generate up to 2.8 Gb of data, enabling direct, real-time DNA and cDNA sequencing. The MinION flow cells are compatible with both MinION and GridION and is a desktop sequencer that allows to run five independent flow cells simultaneously, generating up to 50 Gb of data for sequencing DNA, cDNA, or native RNA in real time. Finally, the PromethION cells are compatible only with the PromethION platform and can generate up to ∼300 Gb for sequencing DNA, cDNA, or native RNA in real time.

Nowadays, routine human genome sequencing at a population scale is boosted using the commercially available PromethION sequencer. The PromethION sequencer is designed to run up either 24 or 48 flow cells at a time, allowing for the sequencing of 20–30X human genome coverage per flow cell.

### Raw data analysis

Since the release of the MinION sequencer in 2014, the number of bioinformatics tools used to analyze ONT data has increased significantly. The reads with no limits in terms of length produced by ONT could offer a promising platform to investigate SVs for medical genetics. Single ONT reads frequently reach hundreds of kilobases in length, with a current record of over 4 Mb, allowing to encompass large SVs end-to-end in a single read. The three main phases that are usually utilized for the identification of SVs comprise the quality control of the sequencing run, alignment of the high-quality reads to the human reference genome, and variant calling.

MinKNOW, the ONT proprietary software application, is used to set the sequencing parameters such as the sequencing time, sequencing library preparation kit, basecalling options, output of the run, and quality cutoff for FASTQ files.

The first versions of MinKNOW outputted each fast5 file (single-fast5), a nested file structure based on file-directory-like construction, for each single read, while the later version generates one fast5 file for 4,000 reads (multi-fast5). When MinKNOW runs in the basecalling mode, it generates both fast5 and FASTQ files. Several third-party algorithms were developed to carry out tasks related to quality control, data exploration, and visualization (e.g., Poretools and PyPore) (Loman and Quinlan, 2014; Semeraro and Magi, 2019).

Basecalling is a crucial step for the nanopore sequencing workflow as it allows the conversion of a raw current signal into a string of nucleotides. The first nanopore basecalling algorithm was provided on the Metrichor cloud and was based on the hidden Markov model (HMM). Metrichor for the R7.3 version of the flow cell recognized the electric signal from 6-mers. Since ONT grew rapidly and basecalling algorithms developed dynamically, most of them are deprecated (e.g., Metrichor and Albacore). Wick et al. (2019) compared four basecalling algorithms developed by ONT, namely, Albacore, Guppy, Scrappie, and Flappie, and a third-party basecaller, Chiron (Teng et al., 2018). They concluded that Guppy performs best in terms of both read and consensus accuracy. Table 3 shows the features of 10 basecallers specifically developed for nanopore sequencing (Makałowski and Shabardina, 2019).

MinION, GridION, and PromethION software applications are provided with Guppy, and basecalling is carried out in real time locally on the machine after a run has finished or a combination of the two.

The latest version of Guppy integrates three different basecalling models: a fast model, high-accuracy (HAC) model, and super-accurate (SUP) model.

These three models differ mainly in terms of accuracy and computational effort. In particular, the fast model is the fastest and the least computationally intense at the cost of the lowest accuracy (<90%) compared with the others. The fast model shows the highest compatibility with real-time basecalling on nanopore devices. The HAC model provides a higher raw read accuracy than the fast model (∼90%) and shows intermediate speed and computational requirement compared, on the one hand, with the fast model, and on the other hand, with SUP. The SUP model is the most accurate (∼99.5%) and is even more intensive than the HAC model. The SUP model shows lower compatibility with the real-time basecalling device. Despite the differences in accuracy, all these models are suitable for unraveling genomic variants (SVs and SNVs) and for genomic phasing and assembly.

In addition to fast5 files, for each sequencing experiment, the sequencing summary file and FASTQ files, split into passed FASTQ files and failed FASTQ files based on the quality control threshold set by MinKNOW, are generated. fast5 and the sequencing summary file could be used for quality control assessment of nanopore sequencing run using Summary Statistics and QC tutorial (https://github.com/nanoporetech/ont_tutorial_basicqc) and NanoR (Bolognini et al., 2019), respectively. While Summary Statistics and QC tutorial use the R markdown and the sequencing_summary file from Guppy basecalling software, NanoR starts either from basecalled FAST5 or a combination of sequencing summary and FASTQ files. Nanoplot, developed by de Coster et al. (2018), performs quality control analysis, starting from both raw FASTQ files and aligned SAM/BAM files.

Alignment is the step used to provide the precise location in the genome of each base pair in each sequencing read. The FASTA/FASTQ reads were aligned to the human reference genome, either GRCh37, GRCh38, or the new T2T-CHM13 (Nurk et al., 2022), using one of the alignment tools available.

Several aligners have been developed with the aim to reduce the error-prone characteristics of a long read. GraphMap was the first aligner algorithm specifically developed for ONT (Sović et al., 2016). Thereafter, the Burrows–Wheeler Aligner (BWA) was tuned to work with nanopore data using the BWA-SW algorithm. Historically, BWA represented the software application of choice to align reads using the short-read sequencing platform (Li and Durbin, 2010). Li extended the BWA-MEM (maximal exact match) algorithm by combining relaxed scoring of the Smith–Waterman algorithm with heuristics filtering to support long and high-error rate sequences from long-read sequencing.

minimap2 (Li, 2018) stands out as the current aligner of choice for long reads, such as NGMLR (Sedlazeck et al., 2018), GraphMap, LAST (Kiełbasa et al., 2011), and LAMSA (Liu et al., 2017), among others; it is faster than the existing long read aligners and shows a precision superior to the others (Gamaarachchi et al., 2019). The hash table-based approach in minimap2 is efficient for the alignment of long reads, while FM-index aligners, such as BWA (Li and Durbin, 2009) and Bowtie (Langmead et al., 2009), were shown to be less efficient with ultra-long reads (i.e., several hundred kilobases or more). Zhou et al. (2019) evaluated the performance of four aligners, minimap2, NGMLR, GraphMap, and LAST, in terms of computing time, maximum memory usage, and file system operations. The work confirmed that minimap2 is the fastest and GraphMap is the slowest aligner and that the consumption of memory (in bytes) is comparable among minimap2, NGMLR, and LAST, while GraphMap consumed the largest amount of memory (Table 1).

**TABLE 1:** Tools specifically developed to handle the alignment and structural variant calling on nanopore data.

Tool	Description	Input	Algorithm	Link	Reference
GraphMap	Aligner	FASTA/FASTQ	Global alignment	https://github.com/isovic/graphmap	Sović et al. (2016)
BWA	Aligner	FASTA/FASTQ	BWA-SW	https://github.com/lh3/bwa	H. Li and Durbin (2010)
minimap2	Aligner	FASTA/FASTQ	Seed-chain-align	https://github.com/lh3/minimap2	H. Li (2018)
NGMLR	Aligner	FASTA/FASTQ	Convex scoring model	https://github.com/philres/ngmlr	Sedlazeck et al. (2018)
LAST	Aligner	FASTA/FASTQ	Adaptive seed	https://github.com/mcfrith/last-genome-alignments	Kiełbasa et al. (2011)
LAMSA	Aligner	FASTA/FASTQ	SDP-based algorithm	https://github.com/yangao07/LAMSA	Liu et al. (2017)
LRA	Aligner	FASTA/FASTQ	Heuristic finding approximate local alignment	https://github.com/ChaissonLab/LRA	Ren and Chaisson (2021)
Sniffles	SV-caller	BAM	Putative variant scoring. Variant scoring and genotyping. Clustering and nested SVs	https://github.com/fritzsedlazeck/Sniffles	Sedlaeck et al. (2018)
Sniffles2	SV-caller	BAM	Three-phase clustering process	https://github.com/fritzsedlazeck/Sniffles	Sedlazeck et al. (2018), Smolka et al. (2022)
SVIM	SV-caller	FASTQ/BAM	Collection of SV signatures. Clustering of the SV signatures. Combination and classification of the signatures	https://github.com/eldariont/svim	Heller and Vingron (2021)
NanoVar	SV-caller	BAM	Artificial neural network model	https://github.com/benoukraflab/NanoVar	Tham et al. (2020)
NanoSV	SV-caller	BAM	Clustering of split reads to identify SV breakpoint junctions	https://github.com/mroosmalen/nanosv	Cretu Stancu et al. (2017)
cuteSV	SV-caller	BAM	Heuristic method	https://github.com/tjiangHIT/cuteSV	Jiang et al. (2020)
Nano-GLADIATOR	CNV-caller	BAM	Read count	https://sourceforge.net/projects/nanogladiator/	Magi et al. (2019)
QDNAseq	CNV-caller	BAM	Read count	https://github.com/ccagc/QDNAseq	Scheinin et al. (2014)

Recently, Ren and Chaisson (2021) developed a long-read aligner that implemented sparse dynamic programming with a concave-cost gap penalty to increase sensitivity and specificity for SV discovery. The memory consumption of LRA (13.96 G) is less compared to that of minimap2 (22.88 G) and NGMLR (17.00 G); moreover, this alignment approach increases sensitivity and specificity for SV discovery from ONT data.

## Overview of the structural variants calling methods

The discovery of genetic variants is the process that enables the identification of the differences between a dataset and a reference genome. Such alterations (call) are reported as SNVs and indel or more complex variants as SVs, including large deletions and insertions, inversions, duplications, and translocations. Several tools were developed for structural variant calling to specifically fit with long-read data, including Sniffles (Sedlazeck et al., 2018), SVIM (Heller and Vingron, 2021), NanoVar (Tham et al., 2020), NanoSV (Cretu Stancu et al., 2017), and cuteSV (Jiang et al., 2020). The algorithm used by each of these SV-callers is summarized in Table 1.

The Genome in a Bottle (GIAB) and Human Genome Structural Variation (HGSV) consortia released high-coverage nanopore sequencing data with high-quality SV callset, enabling an accurate estimation of precision and recall of SV calling software in 2019 and 2020 (Chaisson et al., 2019; Zook et al., 2020).

de Coster et al. (2019) compared germline structural variants calling tools in the genome of the Yoruban reference individual NA19240 using the long-read sequencing platform Oxford Nanopore PromethION. SVs were called using Sniffles, NanoSV (Cretu Stancu et al., 2017), and SVIM.

Sniffles first estimates the distribution in alignment scores and distances between indels and mismatches on the read, as well as the ratios of the best and second-best alignment scores, then scans the read alignments, and segments to determine if they potentially represent SVs. Any potential SVs are clustered and scored based on the number of supporting reads, the type and length of the SV, consistency of the SV composition, and other features. Sniffles can optionally genotype the variant calls and provide clustering of SVs based on the overlap with the same read (Sedlazeck et al., 2018). Sniffles was ultimately implemented in Sniffles2 to accurately detect germline, somatic, and population-level SVs, starting from nanopore data (Smolka et al., 2022). The major novelty introduced using Sniffles2 is the ability to detect low-frequency/mosaic SVs while maintaining high precision.

SVIM analyzes one read at a time and extracts signatures of SVs from SAM/BAM files, and in particular, it searches for two types of signatures: intra-alignment signature (large gap in the reference or in the read) and inter-alignment signature (discordant alignment positions and orientation of the alignment segments of the read). After being collected, signatures are merged by combining the graph-based clustering approach with a novel distance metric for SV signatures. Then, the SV signature clusters are combined to classify events into five distinct classes: deletions, inversions, novel element insertions, tandem duplications, and interspersed duplications (Heller and Vingron, 2021).

NanoSV uses clustering of split reads to identify SV breakpoint junctions. First, all mapped segments of each split read are ordered based on their positions within the originally sequenced read. The aligned read may contain gaps, defined as read segments, which are either unaligned or segments that fail to reach the mapping quality threshold Q1 (default: 20). Evidence from different reads that support the same candidate breakpoints is further collected by clustering all candidate breakpoint junctions that have the same orientation and have start and end coordinates that are in close genomic proximity. For NanoSV variants, the genotype is assigned based on Bayesian likelihood (Cretu Stancu et al., 2017).

de Coster et al. evaluated the aligners LAST, NGMLR, and minimap2 in combination with the SV callers NanoSV, SVIM, pbsv, and Sniffles to regrade precision, recall, and F-measure (harmonic mean of precision and recall) for the genome NA19240 and found that Sniffles in combination with NGMLR or minimap2 alignment achieved the highest F-measure. Sniffles after NGMLR alignment reached the highest precision, while with minimap2, it reached the highest recall. SVIM appeared to be insensitive to different aligners and reached the highest recall, at the expense of lower precision than Sniffles. Compared with Sniffles and SVIM, NanoSV appears to be slow in terms of performance. The principal limitation of NanoSV is that software optimization requires handling large volumes of data and limits runtime and memory usage. The authors executed NanoSV per chromosome in parallel to keep the runtime reasonable, with the limitation that inter-chromosomal variants cannot be detected. Although LAST (Kiełbasa et al., 2011) is the recommended aligner for NanoSV, the SV accuracy seems to be largely independent of the aligner used.

Furthermore, the combination of SVIM and Sniffles after minimap2 alignment was the fastest combination, reaching a recall of 76% and a precision of 51%, being almost as sensitive as the combination of all callers after NGMLR alignment but with greater precision (de Coster et al., 2019).

Recently, Bolognini and Magi (2021) performed a comprehensive evaluation in terms of precision, recall, and F-score of five SV callers (Sniffles, SVIM, cuteSV, npInv, and pbsv) across four long-read aligners (minimap2, NGMLR, LRA, and pbmm2) using both real and simulated ONT data. This comparison was made on ONT PromethION data released by the GIAB consortium for the NA24385 Ashkenazim individual introducing cuteSV compared to the others already evaluated by de Coster (Shafin et al., 2020). CuteSV can detect SVs through multiple steps composed by the collection of SV signatures, the clustering-and-refinement approach to precisely distinguish the SV signatures from heterozygous SVs, and the genotyping of SVs based on the refined clusters of SV signatures (Jiang et al., 2020).

According to de Coster, the highest precision at a cost of low recall was obtained using Sniffles in combination with NGMLR. In this setting, most of the false-negative SVs were shorter than 500 bp. In contrast, Sniffles achieved the lowest F-score compared with cuteSV, SVIM, and pbsv. On the one hand, considering the SV types, cuteSV, SVIM, and pbsv had the best performances in the detection of deletions; on the other hand, cuteSV after NGMLR or minimap2 showed the highest F-score for the detection of insertions and duplications, together with the SVIM–NGMLR combination. All the software applications considered performed well in identifying inversions, while SVIM–minimap2 and pbsv performed better than the other aligner–SV caller combinations for the identification of translocation breakpoints. They further evaluated the genome coverage dependencies of the SV caller, identifying a threshold of ∼20X to achieve the best precision. Sniffles is recommended when considering the highest precision, while cuteSV and SVIM perform the best in terms of recall. Given these results, they concluded that a minimum of five reads supporting SVs could be a good tradeoff to reduce both false-negative and false-positive rates. Overall, they recommended the combination of cuteSV, Sniffles, and SVIM to reduce the final false-positive rates. Recent data suggest that at least 5–10 reads supporting a call should be collected for a good tradeoff to optimize precision and recall (Bolognini and Magi, 2021).

Tham et al. (2020) developed a novel SV caller utilizing low-depth (8X) whole-genome sequencing data generated by ONT and artificial neural network (ANN) inferencing from a simulation-trained model. As for the other tools, NanoVar envisions several steps which include long-read sequence mapping, SV characterization by read-depth calculation, and ANN. HS-BLASTN is used for the alignment step; it is based on a faster MegaBLAST algorithm (Chen et al., 2015). The read depth is calculated at each break-end for SV-associated reads and normal reads separately. Finally, a trained ANN model is employed to improve SV characterization accuracy by evaluating read alignment characteristics and break-end read depth information.

The detection of a large CNV is based on the read depth; therefore, an excess of coverage is evidence of amplification, and conversely, a loss of coverage is suggestive for deletions.

Magi et al. (2019) developed a novel software tool, Nano-GLADIATOR, which can perform CNV detection and allelic prediction of nanopores on WGS data. Nano-GLADIATOR relies on the read count (RC) approach and its correlation with local GC content and mappability to obtain noisy signals in which deletions or duplications are intended as the decrease or increase in RC across multiple consecutive windows.

Scheinin et al. (2014) presented QDNAseq, an R package developed to perform segmentation and CNV calling. Although this algorithm was originally developed using formalin-fixed paraffin-embedded (FFPE) materials, it was recently introduced in the official “human variation workflow” of Nanopore EPI2ME Labs (https://github.com/epi2me-labs/wf-human-variation). This workflow was developed to analyze variation in human genomic data and detect small variants as well as SVs and CNVs.

QDNAseq exploits fixed-sized bins to calculate annotation data (e.g., GC content and mappability), thus facilitating computation and analysis procedures. QDNAseq determines the copy number status of bins (1, 5, 10, 30, 50, 100, and 1,000 Kb) based on the read depth strategy, which correlates the copy number of a region with the depth of coverage (https://labs.epi2me.io/copy-number-calling-update).

A throughout benchmark of available strategies for the identification and characterization of CNVs from nanopore data is greatly needed.

## Structural variants in cancer

It has been demonstrated that the onset and progression of cancer could be triggered by the accumulation of structural abnormalities in the genome as the result of increased genome instability. Somatically acquired SVs could lead to cancer onset by deactivating tumor suppressor genes and upregulating oncogenes. The detection and classification of these variants could improve our understanding of pathologic mechanisms and ameliorate diagnosis, prognosis, and therapy strategies for cancer patients (Hayes, 2019). Given the nature of SVs, it should be pointed out that such complex rearrangements are an important source of variation, accounting for the greatest number of altered bases across the whole genome (Alkan et al., 2011; Sudmant et al., 2015).

Accumulating evidence (Table 2) suggests that SVs can contribute to oncogenesis through multiple mechanisms, including the aberrant activation of oncogenes and inactivation of tumor suppressor genes by translocations, amplifications, deletions, or inversions.

**TABLE 2:** Recent findings using nanopore sequencing and the related findings in cancer.

Sequencing technology	Cancer	Focus/finding	Reference
ONT—MinION	Pancreatic cancer	Large deletions, inversions, and translocations led to the inactivation of CDKN2A/p16 and SMAD4/DPC4	Norris et al. (2016)
ONT—MinION	Liver cancer	Identification of polymorphic and somatic SVs	Fujimoto et al. (2021)
ONT—MinION and PromethION	Hereditary cancer syndrome	Long-read sequencing improves the validation, resolution, and classification of germline SVs	Thibodeau et al. (2020)
ONT—GridION	Acute myeloid leukemia	Development of short-molecule nanopore sequencing for sensitive and accurate detection of CNVs in AML	Baslan et al. (2021)
ONT—MinION	Cell-free DNA lung cancer	Whole-genome molecular karyotypes of six lung cancer types	Martignano et al. (2021)

In pancreatic cancer, a series of SVs, including large deletions, inversions, and translocations, led to the inactivation of *CDKN2A*/*p16* and *SMAD4*/*DPC4*. Norris et al. (2016) identified and characterized 10 SVs (two interstitial deletions, four translocations, four inversions, and one combination of an inversion and translocation), providing proof of principle of the potentiality of nanopore sequencing to detect SVs that resulted even correctly and reliably with a sample dilution of 1:100. A 450X coverage in the region of interest is required to achieve 99% of confidence at 1:100, thus limiting the identification of rare SVs to a targeted sequencing approach.

The genomic landscape of MM is mainly characterized by recurrent SNVs and CNVs, most of which were identified by WES and array-based technologies. The first studies that aimed to characterize the SV landscape in MM were usually limited to translocations involving *MYC* gene (Bolli et al., 2018; Barwick et al., 2019). Recently, the whole-genome sequencing approach was utilized in this context to shed light on the genomic complexity of MM, demonstrating a pivotal role of SVs in the development of such neoplasia. Rustad et al. (2020) reported the first comprehensive study of SVs in MM patients exploiting ONT. The authors identified three main patterns of SVs, chromothripsis, templated insertions, and chromoplexy, overall suggesting SVs as the missing piece to understand the driver landscape of MM. A total of 68 SV hotspots were identified, of which 53 were not previously reported. The characterization of the SV breakpoints revealed 17 new potential driver genes, among which *TNFRSF17*, *SLAMF7*, and *MCL1* were the most relevant for their potential therapeutic impact. Moreover, chromothripsis was detectable in 24% of newly diagnosed patients, providing a rationale for including this phenomenon in the clinical risk score.

ONT application was also exploited in liver cancer patients, with the aim to obtain a comprehensive landscape of both germline and somatic SVs by which the biological mechanism of SV generation was inferred. Taking advantage of long-read sequencing technology to resolve SVs, the novel algorithm CAMPHOR (https://github.com/afujimoto/CAMPHOR and https://github.com/afujimoto/CAMPHORsomatic) showed that most of the insertions were caused by transposons *Alu* and LINE. Overall, 106 polymorphic tandem duplication candidates (74 detected from insertions and 32 from intra-chromosomal translocations), 15 polymorphic template sequence insertion candidates, and 15 polymorphic insertions of processed pseudogenes were found. As for other sequencing technologies, these findings suggest that the use of both germline and somatic samples boosts the resolution of the complex structure in cancer genomes (Fujimoto et al., 2021).

Recently, long-read sequencing was used to investigate germline variants in cancer predisposition and susceptibility, aiding in classifying those SVs that were not resolved by NGS. Thibodeau et al. (2020) identified 12 germline SV candidates: eight deletions, two inversions, and two complex rearrangements (three or more breakpoints). In one sample, they identified a novel complex rearrangement on chromosome 5q35, where a 194 kb inverted duplication was flanked by a small indel. In another sample, they found an 85 kb inversion with breakpoints in *TSC2* and *TRAF7* flanked by two deletions on chromosome 16p13.3, resulting in the partial loss of *NTHL1* and *TSC2*. Moreover, long-read sequencing resolved an inversion in RAD51C that was missed by the Illumina short-read sequencing platform. Overall, their findings suggest that 1.5% of the cases were highlighted by cancer susceptibility induced by SVs.

Cancer genomes are shaped by chromosome instability, leading to the acquisition of somatic CNVs that ultimately model the diagnostic, prognostic, and therapeutic approaches; therefore, inferring CNVs is of clinical relevance. This is particularly true in those pathologies dealing with extreme mutation heterogeneity, such as for acute myeloid leukemia.

Since the typical number of reads obtained by a single run by whole-genome sequencing approaches is limited, one could target specific genomic regions in order to increase the number of reads obtained, resulting in higher sensibility. Baslan et al. (2021) optimized the nanopore workflow to overcome the limitation caused by the low number of sequencing reads by loading short molecules of DNA (median read length ∼500 bp). This approach showed a 4–6-fold increase in the number of reads; moreover, the results are fully concordant with those obtained by standard NGS and at a much higher resolution than conventional karyotyping. In this study, ONT data were successfully used to detect ∼10 Mb deletions on chromosomes 5 and 7, complex rearrangements involving chromosome 11 resulting in *MLL1*/*KMT2A* gene fusions, and focal alterations on 12p and 16p. Nanopore data enabled a copy number profile with greater informativeness than standard cytogenetics, yielding high-quality reads per molecule count. The high quality of the reads generated by ONT allowed the identification of cryptic CNVs, such as a focal deletion on 17p encompassing the *TP53* gene, as well as other alterations involving *MLL* and *TP53* that were confirmed by FISH.

One of the first reports on nanopore sequencing with cell-free DNA (cfDNA) was published by Martignano et al. (2021), who exploited long-read sequencing to profile the CNVs of tumor patients using plasma samples. This approach allows to monitor the tumor evolution at different time points with limited harm and risk for patients. The results demonstrated the same performance in terms of CNV detection for Illumina and ONT; however, the lower cost of the latter has the potential to make ONT approaches more widely accessible. Moreover, the real-time analysis allowed by nanopore can obtain results (from blood withdrawal to bioinformatics analyses) within 1 day. Conversely, NGS approaches, based on sequence-by-synthesis technologies, make reads available only at the end of the whole run, which can last some days.

## Structural variants in prenatal diagnostic testing

Chromosome alterations, such as aneuploidy and hotspot SVs, constitute the major cause of stillbirth, fetal structural abnormalities, and intellectual disabilities (Table 3). Consequently, prenatal diagnostic testing mostly relies on the identification of those genetic alterations. Conventional testing methods are based on two main approaches: the rapid and targeted approach and the slow whole-genome approach. Rapid and targeted techniques, including FISH, multiplex ligation-dependent probe amplification, and quantitative polymerase chain reaction assays, are limited to a specific subset of chromosomes. Conversely, whole-genome approaches, including G-banded karyotyping, chromosomal microarray analysis, and next-generation sequencing, interrogate the entire genome, but they need days to week to complete. As in many other areas of biomedical research, in prenatal diagnostic testing, TGS may enable easier and more informative analysis of genetic variants, providing new insights into the mechanism of various diseases (Lin et al., 2021). Wei et al. (2022) developed an ultra-rapid approach for library preparation, sequencing, and data analysis that enables the screening of prenatal aneuploidy. Such a nanopore-based approach allowed the on-site testing assessment of aneuploidy for all chromosomes on the same day. Similarly, Bartalucci et al. (2019) provided proof of principle of the feasibility of nanopore-based karyotyping to detect and characterize SVs, starting from the chorionic villus or amniotic fluid. The approach used Nano-GLADIATOR (Magi et al., 2019) to detect both aneuploidies and SVs of limited size, such as the 1 Mb deletion involving 22q related to DiGeorge syndrome found in one sample. These results were obtained in less than 72 h at an affordable cost.

**TABLE 3:** Recent findings using nanopore sequencing and the related findings in prenatal diagnostic testing.

Sequencing technology	Focus/finding	Reference	Year
ONT—MinION	Development of short-read transpore rapid karyotyping (STORK) for genome-wide aneuploidy detection	Wei et al. (2022)	2022
ONT—GridION	Rapid detection of trisomy 8, 13, and 21 and focal deletion in 22q	Bartalucci et al. (2019)	2019
ONT—PromethION	Resolving of SVs affecting the Prader–Willi syndrome locus	Deest et al. (2022)	2022
ONT—PromethION	Accurate categorization between non-translocation embryos and translocation carrier embryos and precisely localizing the translocation breakpoints	Xia et al. (2023)	2023
ONT—MinION	Parental haplotype reconstruction and characterization of balance or imbalance wild-type and mutant alleles in maternal plasma	Jiang et al. (2021)	2021

In a recent study, ONT was employed to fully characterize the Prader–Willi syndrome (PWS) locus and led to the identification of common SVs in many PWS patients. Moreover, the authors associated a high SV burden with PWS patients affected by schizophrenia and bipolar and autism spectrum disorders. Moreover, they demonstrated that SVs involving OPRM1 and OPRL1 disrupted the opioid system and nociceptin/orphanin systems. A deletion of approximately 6 Mb in chr15q11.2–13, occurring in ∼60% of PWS patients, causes the loss of a cluster of genes. Authors resolved the large deletions of chr15q11.2–chr15q13 on the paternal allele due to long reads generated by ONT PromethION, enabling the determination of the genetic subtypes in PWS in 85% of the cases. In addition to the aforementioned disease-causing alterations, they identified a median of 28,123 SVs mostly located within introns (64.1%) or intergenic locations, whereas only a minority of SVs were located in exons (0.73%), 3′-UTR (0.21%) or 5′-UTR (0.04%). The study pointed out chr22q11.2 as the SV hotspot; it is known by the literature that duplication in this region is associated with an intellectual or learning disability, developmental delay, slow growth leading to short stature, a weak muscle tone, and neuropsychiatric comorbidities, such as those observed in PWS. A novel 65 bp deletion was also found in 3′-UTR of *catechol-O-methyltransferase* (*COMT*), a gene that likely contributes to obsessive-compulsive disorder (OCD), potentially explaining the ritualistic behavior of PWS patients (Deest et al., 2022).

The characterization of translocation breakpoints at a single-base resolution is extremely important in context in which the event disrupts a disease-causing gene. The long-read approach might improve our ability to correctly resolve the breakpoints, avoiding the laborious amplicon-based Sanger sequencing. Considering this, Xia et al. pointed out a full characterization of the translocation breakpoints at a single-base resolution in patients who carried balanced reciprocal translocation (BRT). Recent studies demonstrated the positive association between BRT and clinical diseases such as neurocognitive disabilities and Tourette syndrome (Schluth-Bolard et al., 2013; Nilsson et al., 2017). FISH, single-nucleotide polymorphism (SNP), aCGH, and NGS have been applied to preimplantation genetic testing (PGT) for identifying normal or balanced diploid embryos in BRT carriers. All these technologies show limitations mostly due to the detection of specific chromosomes (FISH) and the inability to distinguish euploid carriers and non-carrier embryos (aCGH and NGS). In this context, ONT correctly identified the breakpoints for the two patients at chr2:125157514–chr5:35465883 and chr13:26208296–chr17:33942282, which were concordant with the “Mapping Allele with Resolved Carrier Status” (MaReCs) results, eventually distinguishing normal embryos from carrier embryos in PGT (Xia et al., 2023).

Haplotype phasing is also of key importance in prenatal diagnostic testing to assess the parental origin of *de novo* alterations. Jiang et al. (2021) used targeted long-read sequencing by ONT to reconstruct the parental haplotypes without a proband sample by sequencing the ∼50-kb targeted region containing the *HBB* gene previously enriched by long-range PCR of 10 and 20 kb. Such methods allowed the analysis of balance or imbalance wild-type and mutant alleles in maternal plasma, enabling the diagnosis of β-thalassemia and the correct haplotype phasing of 12 families.

## Resolving haplotype phasing

Haplotype phasing is referred to as the process by which a genetic variant is assigned to the homologous paternal and maternal chromosome. This process is a crucial step in situations where it is important to understand the inheritance patterns. Many methods for haplotype phasing have been developed; however, until the advent of TGS, they mainly relied on short-read NGS technologies (Browning and Browning, 2011). Given the length of typical long reads, ONT data enable the direct phasing of complex genomic regions, such as the major histocompatibility complex (MHC), avoiding statistical imputation.

The *HLA* regions are the most polymorphic loci in the human genome, making their resolution extremely complex, even more with short NGS reads. Ammar et al. (2015) demonstrated that ONT can resolve both variants and haplotypes of *HLA-A*, *HLA-B*, and *CYP2D6*, which are crucial for determining drug response of patients. The genetic variants were correctly assigned in the absence of parental haplotypes or statistical phasing.

The haplotype resolution is also important for the quality of genome assembly. Genome assembly is referred to as the process of ordering the nucleotides from a longer DNA sequence to reconstruct the original sequence. As for the resolution of the haplotypes, genome assembly could be tricky with the short-read sequencing approaches due to the genome size (∼3.1 Gb), the heterozygosity, the regions with high GC content, diverse repeated elements, and segmental duplications (up to 1.7 Mbp in size), which represent a good proportion of the whole genome. Given that ultra-long reads have the power to facilitate the assembly and phasing of the MHC, Jain et al. (2018) provided the first evidence of a complete assembly and phasing of the MHC obtained in a diploid human genome.

## Resolving disease-causing structural variants

Even today, a precise diagnosis may not always be reached in individuals with suspected genetic conditions in which the putative variants do not fully fit with the phenotype, or no variants are identified in hotspot genes. Several studies pointed out the prominent role of SVs as disease causing not only in cancer but also in non-oncologic diseases, as summarized in Table 4.

**TABLE 4:** Recent findings using nanopore sequencing and the related findings in neurodegenerative disorders.

Sequencing technology	Disease	Focus/finding	Reference	Year
ONT—MinION	Congenital abnormalities	Detecting and mapping the breakpoints of chromothripsis rearrangements	Cretu and Stancu et al. (2017)	2016
ONT—GridION	Autosomal-recessive disorder and X-linked disorder	Target long-read sequencing by adaptive sampling accurately identifies pathogenic structural variants, resolves complex rearrangements, and identifies Mendelian variants undetected by other technologies	Miller et al. (2021)	2021
ONT—PromethION	Parkinson’s disease	Characterization and validation of SVs at Parkinson’s disease risk loci	Billingsley et al. (2022)	2022

Cretu Stancu et al. (2017) provided proof of evidence of the detection of *de novo* chromothripsis rearrangements by exploiting ONT whole-genome sequencing in two patients. Moreover, the long reads provided by ONT were used to phase the genetic variation, allowing the assessment of the parental origin of the rearrangements. They developed a pipeline to extract all known *de novo* breakpoint junctions, and more than 32% of chromothripsis breakpoint junctions were detected using MinION compared to short-insert Illumina sequencing. Overall, more than 14% of the high-confidence SVs identified by nanopore sequencing were not detected in the matching Illumina sequencing, thus demonstrating the advantage provided by long reads in all the research areas where SVs play a pivotal role.

Concerning the detection of the disease-causing variants in a diagnostic setting, a key parameter is represented by the sequencing coverage in target regions. This is particularly true if we consider the relatively high error rate of typical ONT run, largely due to the inability to control the speed of the DNA fragments through the pore. Whole-genome long-read sequencing generates an amount of reads inadequate to resolve complex SVs. To this end, a computational approach, named adaptive sampling, was recently introduced as an alternative to laboratory-based depletion or enrichment to increase the coverage in clinically relevant regions. The adaptive sampling approach allows to selectively sequence the strands mapped to a predefined region of interest based on the real-time alignment of the first nucleotides (200 as default) of each strand flowing through the pore. The sequencing strands whose first portion does not align with the region of interest are ejected by reversing the ionic current through the pores. The decision to sequence or eject the strand is carried out in few seconds (https://community.nanoporetech.com/posts/beta-release-of-adaptive-s-7369) (Martin et al., 2022). This computational method aims to enrich target regions by preserving almost the pore consumption for the sequencing of the selected regions without amplifying them. Adaptive sampling represents an intriguing new method for target sequencing approaches; on the other hand, it needs a careful selection of the size of the regions of interest and of the sequence for real-time alignment. The first adaptive sampling algorithm was developed in 2016 as dynamic time warping (Loose et al., 2016), and although it still needs improvement, this enrichment method has attracted growing interest for its potential to make sample amplification and library preparation dispensable. Adaptive sampling for ONT was initially available through third-party software, and since 2020, it was included in the ONT MinKNOW software as user-selectable options.

Users must provide target and reference files and choose the enrichment or depletion mode. Adaptive sampling was successfully used for the screening of a cohort of patients lacking a precise diagnosis and with no candidate variants to identify pathogenic or likely pathogenic alterations.

As previously discussed, the long reads ease the study of complex structural rearrangements, enabling the resolution of variant breakpoints and the identification of unbalanced translocations. In a certain clinical context, the characterization of the alteration is of relevance to refine the clinical management of the disease, e.g., in genetic disorders. In one patient, ONT sequencing was exploited to characterize the translocation between chromosomes 12q and 17q, known to affect the gene *SOX9* and leading to campomelic dysplasia. The analysis of long reads allowed to precisely map the variant breakpoints 164 kbp far from the *SOX9* locus. This result led to the identification of a novel potential pathogenic region close to the (1 Mb) *SOX9* sequence (Pfeifer et al., 1999; Miller et al., 2021).

In addition to the characterization of known genomic regions, the use of long reads facilitates the discovery of novel variants potentially affecting disease-causing genes. The role of recurrent SVs in Parkinson’s disease is not fully understood; the most recent genome-wide association study (GWAS) aimed to improve the understanding of the disease mechanisms through a deep characterization of common structural variants. Several studies suggest a putative role of such variants in monogenic forms of Parkinson’s disease and parkinsonism. In particular, the partial deletions of *PARK2* and CNV, affecting the whole *SNCA* region, were shown to be causative of autosomal-recessive Parkinson’s disease and autosomal-dominant Parkinson’s disease, respectively. Authors genotyped and tested over 3,000 common SVs identified by previous studies, accounting for ∼400 million nucleotides, and validated the presence of three novel SVs affecting the *PARK2* and *SNCA* regions and a 2 Kb deletion within intron 3 of LRRN4, which was supposed to be a causal variant in the Parkinson’s disease locus (Billingsley et al., 2022).

## Discussion

The latest genome-wide studies revealed that SVs play a prominent role in many types of cancer and genetic diseases, but they remained understudied compared to SNPs and small indels owing to their difficulty to be detected. In this context, long-read sequencing has the potential to ease the screening of wide and complex genomic regions, thus fostering the discovery of novel variants and the diagnostic informativeness of genetic testing. Short-read approaches represent the gold standard method to study SNPs and indels of few bp, although they are inadequate in resolving large and complex SVs. The growing rate of recent publications points out TGS as a breakthrough technology, chosen as the “method of the year 2022” by Nature Methods (Nat Methods, 2023). Among TGS technologies, ONT provides a nanopore sequencer that allows the sequencing of the whole genome at affordable costs. Intriguingly, nanopore sequencing technology enables the direct sequencing of “native” nucleic acids, which allows to overcome the amplification bias and offers the opportunity to directly detect epigenetic modification of the underlying sequence. Such advantages come at the price of lower throughput and higher error rate than those of NGS. Among the ONT sequencers, a PromethION run can provide whole-genome data from genomic DNA at an average depth ranging from ×15 to ×30, which could be exploited for SV calling. Moreover, the throughput and quality of TGS are greatly dependent on the amount and quality of the input sample; therefore, a careful selection of the sample preparation method is of key importance. The collection of software and analytic pipelines that have been developed to specifically deal with ONT data proves the growing interest in this technology and provides a constant refinement of specific analytical steps, such as preprocessing, alignment, assembly, quantification, and error correction. The latter is the main aspect to be considered, particularly when direct sequencing is exploited for genome-wide studies. Among the features included in ONT MinKNOW software, “live basecalling” enables real-time data analysis and has the potential to provide rapid results. Such an ONT characteristic, together with the improvement in data reliability, has great potential in applications in clinical settings, particularly in those cases where rapid genetic information may help select the best therapeutic option. In conclusion, in spite of the initial skepticism and the need for further improvement, nanopore long-read sequencing is emerging as a robust tool to study wide genomic regions and complex alterations that possibly went undetected till now. TGS data require large data storage and computing power; however, they constitute a goldmine of genomic information that will soon expand our knowledge about the human genome.
